# Insights into Carbon Sphere Formation from Glucose and Levoglucosan During Hydrothermal Carbonisation

**DOI:** 10.3390/molecules31081363

**Published:** 2026-04-21

**Authors:** Ance Plavniece, Galina Dobele, Kristine Meile, Vilhelmine Jurkjane, Aivars Zhurinsh

**Affiliations:** Latvian State Institute of Wood Chemistry, Dzerbenes Str. 27, LV-1006 Riga, Latvia; galina.dobele@kki.lv (G.D.); kristine.meile@kki.lv (K.M.); ligms@edi.lv (V.J.); aivarsz@edi.lv (A.Z.)

**Keywords:** hydrothermal carbonisation, carbon sphere, levoglucosan, Py-GC/MS

## Abstract

The decline of fossil fuel resources and the negative impact of fuel combustion on the environment are forcing scientists to develop new technologies for producing functional carbon materials with various useful properties. This work is devoted to a detailed study of the transformations of monosaccharides, glucose and levoglucosan, during hydrothermal carbonization, aimed at the formation of carbon microspheres. Hydrochars were obtained at temperatures of 200, 250, and 300 °C and characterized using SEM, Py-GC/MS, and elemental analysis. Changes in the chemical composition of the liquid phase were studied, depending on the HTC temperature and precursor concentration. Expanded knowledge of microsphere formation enriches information on the mechanism of monosaccharide transformation for the production of new carbon materials through synthesis from inexpensive precursors.

## 1. Introduction

Depletion of fossil fuel reserves, environmental conditions and the need for cost-effective competitive products are becoming two major areas of research today, forcing scientists to develop new technologies for the production of functional carbon materials with various useful properties [1,2,3].

Carbon spheres are spherical carbon-based materials, typically ranging from nanometers to micrometers in size, valued for their high surface area, porosity, and tunable structures and good electrical conductivity. They are widely used in applications including energy storage, catalysis, CO_2_ sequestration, water purification and agriculture, but have only been actively explored in the 21st century [3,4,5].

To obtain carbon-containing materials with specified properties (shape, size, and functional groups), interest arose in the thermochemical process of hydrothermal carbonization (HTC), due to the mild operating conditions (temperature up to 300 °C, self-generated pressure) in a subcritical water environment (the critical point is reached at 374 °C and 22.1 MPa) [6,7,8].

The size and size distribution of carbon spheres can be controlled by synthesis conditions, such as processing temperature, reaction time, starting material concentration, and precursor type. The properties of process water change significantly with increasing temperature under the influence of self-generated pressures. Therefore, its reaction behavior varies greatly within a narrow temperature range. This can considerably influence the environment surrounding the particles, which determines their colloidal stability and associated size characteristics [9,10]. In 2001, Wang et al. reported the synthesis of carbon-containing microspheres of adjustable size (in the range of 0.25–5 mm) by hydrothermal carbonization of sucrose at 190 °C [11]. Therefore, higher temperatures (260 °C) result in larger particles and a more uniform average size.

Among the saccharides that can be used to produce carbon spheres via HTC, glucose is the most promising material, as it is by far the most abundant and inexpensive saccharide available [12]. As an alternative that does not compete with glucose, anhydrosugars derived from lignocellulose, such as levoglucosan, can be utilized. Unfortunately, little research has been devoted to the detailed chemical and structural properties of spherical carbon materials based on hydrochars from other saccharides and anhydrosaccharides, which are formed during the chemical processing of biomass.

The reaction mechanisms of HTC of glucose include hydrolysis, dehydration, polymerization, condensation and aromatization [3,5,13]. Yao et al. [14] investigated the formation mechanism of carbon microspheres during the low-temperature carbonisation of glucose and fructose. They concluded that during hydrothermal treatment, glucose first loses water through an intermolecular condensation reaction, followed by aromatization (carbonation). R. Demir-Chakan and co-authors [15] synthesized spherical mesoporous structures containing an imidazole group using conventional HTC of glucose and tested them as catalysts for Diels-Alder reactions. According to 13 NMR spectra, glucose hydrochar, like lactose, has a polymer-like structure consisting of polyfuran chains [16]. This structure is formed by polymerization or aromatization of furfural aldehyde and/or 5-(hydroxymethyl)-2-furaldehyde molecules, which are formed during dehydration of the saccharide [17].

The main objective of this work is to expand the understanding of the chemical composition and structural characteristics of hydrochars in the process of formation of microsphere-containing carbon materials obtained under various conditions of hydrothermal carbonization of monosaccharides—glucose and 1,6-beta-D-anhydroglucopyranose (levoglucosan).

## 2. Results

The formation of hydrochar structure under low-temperature conditions depends on several parameters. In this study, the influence of glucose (G) and levoglucosan (LG) concentration and temperature was studied, especially their influence on the yield and elemental composition of the resulting hydrochars.

**Figure 1:**
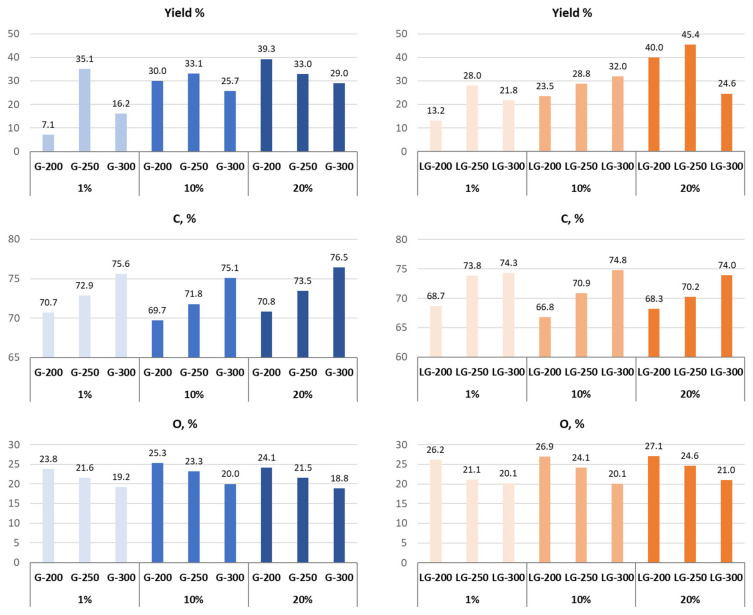
Yield and elemental composition of glucose and levoglucosan at different HTC temperatures (200, 250, 300 °C) and at different precursor concentrations (1%, 10% and 20%).

The dependence of these process parameters can be seen in Figure 1. At concentrations of 1 and 10% for G, the yield of hydrochar increases, reaching maximum values at HTC of 250 °C and then decreasing at 300 °C. The HTC of a 20% solution leads to a gradual decrease in the yield of hydrochar with increasing processing temperature. In the case of LG, the nature of the change in yield for a 1% concentration is the same as for G. With an increase in the concentration of precursor LG to 10%, an increase in yield is observed with increasing temperature, and with an increase in concentration to 20%, the increase occurs only up to 250 °C, after which the yield decreases.

The amount of carbon increases and the amount of oxygen decreases accordingly with increasing temperature for all concentrations of both precursors.

**Figure 2:**
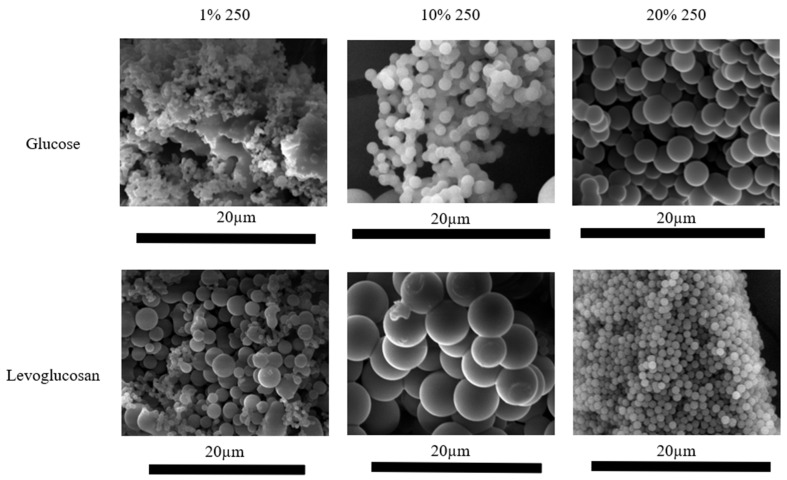
SEM images of hydrochar from glucose and levoglucosan precursor at different HTC temperatures (200, 250, 300 °C) and at different precursor concentrations (1%, 10% and 20%).

Figure 2 shows the morphology of G and LG hydrochars synthesized from 1%, 10% and 20% precursor solutions at HTC temperature 250 °C. For hydrochars from 1% G solution, plate-like particles covered with sphere-like particles are formed. In turn, agglomerates of spherical particles of various sizes are formed from 1% LG solution. Increasing the concentration forms a more ordered structure. For hydrochars from 10% G solution, sphere-like agglomerates begin to form, and for 20% G 250, fully formed sphere agglomerates are visible. A similar situation is for LG samples, only it should be noted that the sizes of the spheres in 20% LG 250 are relatively smaller.

**Figure 3:**
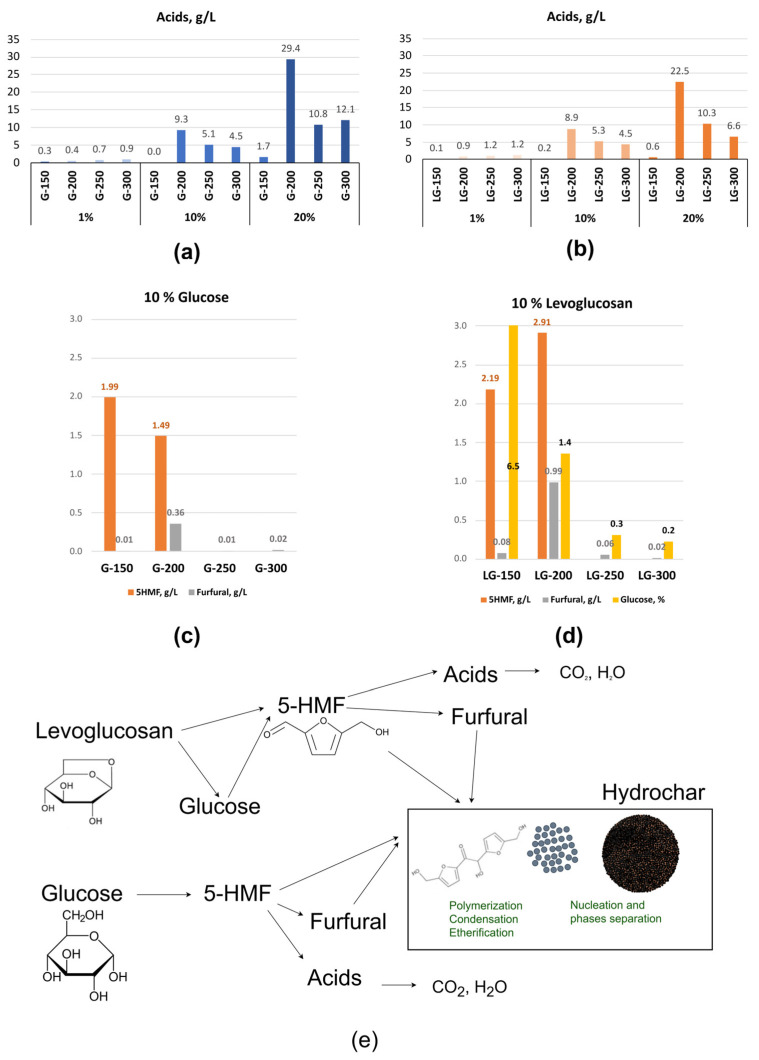
Total acid number (expressed as g/L) content in the HTC process waters content of 10% glucose (**a**) and levoglucosan solutions (**b**); 5-HMF and furfural calculated from UHPLC C18-UV (expressed g/L) content in the HTC process waters of 10% glucose (**c**) and levoglucosan (**d**) solutions; reaction pathway for glucose and levoglucosan solutions (**e**).

Process water was collected for all concentrations at HTC temperatures of 150, 200, 250 and 300 °C. The collected process water samples were further analyzed for total acid number, 5-HMF and furfural determination as well as for reaction pathway generation (Figure 3).

Thus, by varying the HTC temperature and the precursor and its solution concentration, both the particle diameter and size distribution can be controlled. The final chemical structure of the hydrochar ranges from polyfuran to spherical agglomerates with an aromatic structure [17].

Among the many reactions occurring in the HTC process, the main ones are hydrolysis, dehydration, decarboxylation, nucleation, condensation and aromatization [18]. As a result of these reactions, the presence of water-soluble intermediates was established in the aqueous phase, which then condensed and polymerized, forming the hydrochar structure. The scientific literature indicates that significant amounts of 5-hydroxymethylfurfural (5-HMF), levulinic and formic acids, and dihydroxyacetone were detected in the aqueous phase [16]. Phenolic compounds were not detected [12].

Even at a low HTC temperature (150 °C), the primary sugar dehydration product 5-HMF was detected in the aqueous phase for both precursors at 10% concentracion, with its content amounting to 1.99% and 2.19% for glucose and levoglucosan, respectively (Figure 3). In the case of LG, a significant amount of glucose was also detected in the solution (HTC 150 °C), the presence of which indicates an initial hydrolysis reaction for LG. In parallel with this reaction, with an increase in temperature to 200 and 250 °C, the dehydration reaction continues for both glucose and LG, forming 5-HMF, whose further dehydration leads to the formation of furfural. For LG, at HTC temperature of 200 °C, the content of 5-HMF and furfural is higher than for glucose, apparently due to both direct transformation to 5-HMF and the initially formed glucose. The literature also indicates the possibility of forming levoglucosenone by dehydration of LG, with subsequent isomerization, of which a high yield of 5-HMF can be obtained in a two-phase solvent system [19]. With a further increase in temperature, the content of furan compounds decreases due to intense nucleation, condensation, and polymerization.

In the case of LG, when more 5-HMF is released, which further decomposes into furfural and acids, relatively less acid is formed at low HTC temperatures (150–250 °C). As the HTC temperature increases, the resulting acids rapidly decompose into CO_2_ and H_2_O [20]. Such changes affect not only the liquid phase but also particle growth and morphology. Accordingly, an increased amount of acid in process water can promote nucleation with subsequent condensation and phase separation, as well as particle growth [21,22]. As previously discussed in Figure 2, in the LG sample, which contains relatively less acid in the HTC 20% solution, the hydrochar particles have a smaller diameter.

**Figure 4:**
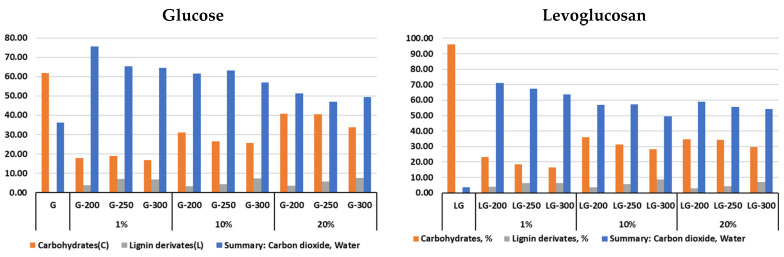
Analytical pyrolysis (Py-GC/MS) of glucose and levoglucosan and hydrochar at different HTC temperatures (200, 250, and 300 °C) and from different feedstock solution concentrations (1%, 10% and 20%), (amount of unidentified compounds 10%).

According to the analytical pyrolysis results (Figure 4), the volatile products of the hydrochar samples after hydrothermal carbonization, compared to the initial glucose and levoglucosan samples, exhibit the highest content of the fraction of highly volatile compounds—water, carbon dioxide, and formaldehyde. For the initial glucose sample, the content of this fraction is almost half as much, while for levoglucosan, it is only 3.5%. With increasing HTC temperature and increasing sample concentration, the content of highly volatile compounds decreases. A characteristic feature is the higher content of highly volatile compounds in glucose hydrochar compared to levoglucosan at all studied HTC temperatures, likely due to the C1-O-C6 bond in the structure.

With an increase in the concentration of precursors, the content of carbohydrate derivatives in the volatile products of hydrochars decreases, and the content of phenolic compounds increases with an increase in the HTC temperature.

For comparison and objective assessment of the formation of individual organic compounds in the groups of carbohydrate and phenolic compounds, the predominant group of highly volatile compounds, including mainly water, was excluded from the calculations.

**Figure 5:**
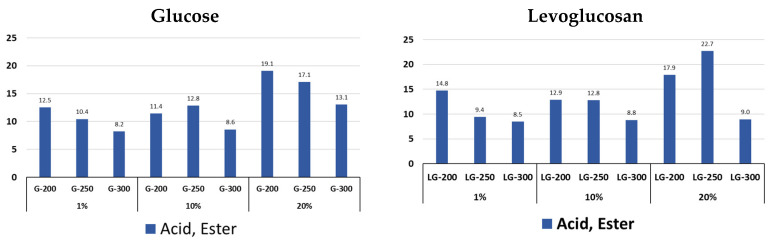
Content of acids and esters in pyrolysis volatile products of glucose and levoglucosan hydrochars at different HTC temperatures (200, 250, and 300 °C) and at different precursor concentrations (1%, 10% and 20%).

Different precursor concentrations and the HTC temperature affect the content of acidic and ester compounds in the volatile products of hydrochar pyrolysis (Figure 5). This shows that both parameters influence the chemical composition of hydrochar. When using concentrations of 1 and 10%, with increasing HTC temperature, the content of this group of compounds similarly decreases for G and LG. Increasing the concentration of the substance to 20% significantly increases the content of compounds of this group for G and LG, with the maximum being reached at 200 °C for glucose hydrochars and at 250 °C for levoglucosan.

The main dominant compounds in this group are acetic, propanoic, and levulinic acids (Table S1). The acetic acid content decreases with increasing HTC temperature and the concentration of both glucose and levoglucosan solutions, while the levulinic acid content increases.

**Figure 6:**
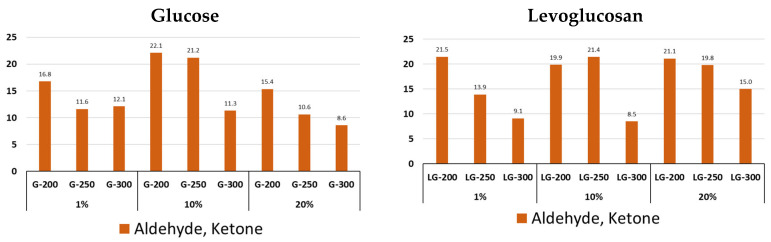
Content of aldehyde and ketone in pyrolysis volatile products of glucose and levoglucosan hydrochars at different HTC temperatures (200, 250, and 300 °C) and at different precursor concentrations (1%, 10% and 20%).

The change in the content of aldehyde and ketone compounds occurs similarly for G and LG—their content decreases with an increase in processing temperature from 200 to 300 °C (Figure 6). It should be noted that the content of this group is higher for LG, with a concentration of 20% at all HTC processing temperatures. The main components of this group are methylglyoxal, 5-hexen-2-one, and 2,5-hexanedione (Table S2).

**Figure 7:**
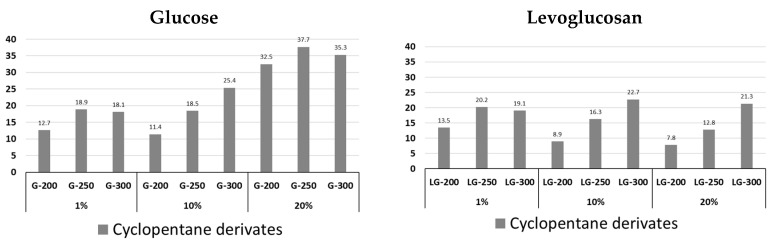
Content of cyclopentane derivative in pyrolysis volatile products of glucose and levoglucosan hydrochars at different HTC temperatures (200, 250, and 300 °C) and at different precursor concentrations (1%, 10% and 20%).

The total content of cyclopentane derivatives (Figure 7, Table S3) for precursor solution concentrations of 1 and 10% increases for glucose and levoglucosan with increasing processing temperature. For levoglucosan, a change in concentration of 20% does not change the quantitative patterns, and the main representatives of these changes are methylene derivatives of cyclopentane—2-Cyclopenten-1-one, 3-methyl- and 2-Cyclopenten-1-one, 2,3-dimethyl. In the case of a GI concentration of 20%, a significant content of 2-Cyclopenten-1-one, 3-(1-methylethyl)- is observed, the relative content of which in the group at 200 °C is 70.6% (Table S3). With an increase in the processing temperature, the content of this compound decreases to 51.2%. During the pyrolysis of LG hydrochar, this compound is also formed, but its content increases with an increase in concentration and temperature only to 10.1%.

**Figure 8:**
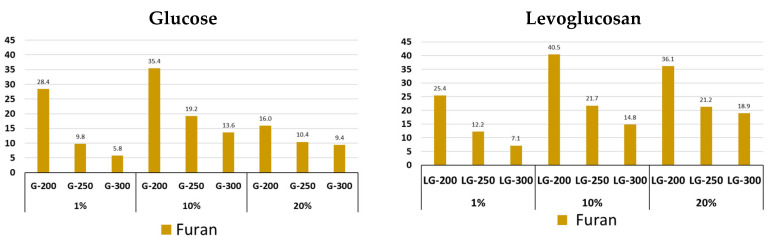
Content of furan derivative in pyrolysis volatile products of glucose and levoglucosan hydrochars at different HTC temperatures (200, 250, and 300 °C) and at different precursor concentrations (1%, 10% and 20%).

The highest content of derivatives of a large group of furan compounds of varying structure (Figure 8, Table S4) for all glucose and levoglucosan concentrations is observed at the lowest HTC of 200 °C and decreases by 2–3 times with increasing temperature. Some furan derivatives are not detected even in small quantities at an HTC of 300 °C. The highest content of furan compounds is observed when using 10% as G, with the dominant compounds of this group being methyl derivatives of furan, as well as 2-furancarboxaldehyde, 5-methyl-. Acetofuran and benzofuran are formed during the pyrolysis of G and LG hydrochars, and their content increases with increasing temperature.

**Figure 9:**
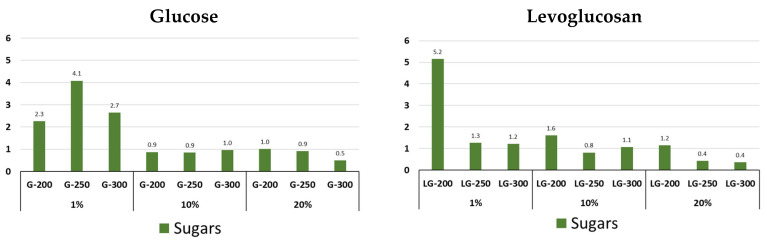
Content of sugar derivative in pyrolysis volatile products of glucose and levoglucosan hydrochars at different HTC temperatures (200, 250, and 300 °C) and at different precursor concentrations (1%, 10% and 20%).

Sugar derivative groups’ main representative, the LG (Figure 9, Table S5), content in hydrochars drops dramatically for both precursors with increasing HTS temperature. The presence of levoglucosan in the hydrochar structure is detected only at 1% precursor concentration, and the maximum amounts for G and LG are 4% (250 °C) and 5% (200 °C). With increasing precursor concentration and HTS temperature, the levoglucosan content does not exceed 1%.

**Figure 10:**
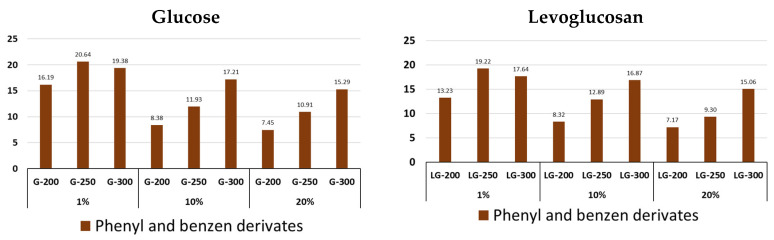
Content of phenyl and benzen in pyrolysis volatile products of glucose and levoglucosan hydrochars at different HTC temperatures (200, 250, and 300 °C) and at different precursor concentrations (1%, 10% and 20%).

The increase in the formation of phenyl- and benzen-aromatic derivatives (Figure 10, Table S5) during hydrochar pyrolysis depends primarily on the low-temperature thermal cycle temperature, and this process is similar for both glucose and levoglucosan. The highest aromatic content in hydrochar structures is observed when using 1% precursor solutions. Phenol, its dimethyl derivative, and benzenemethylethanol predominate among the aromatic compounds.

In addition to these compounds, aliphatic, aromatic and cyclic monomers were found in the volatile pyrolysis products, among which the highest content was formed of 1H-Inden-1-one, 2,3-dihydro-, 7-Methylindan-1-one and 1-Naphthalenol (Table S6).

**Figure 11:**
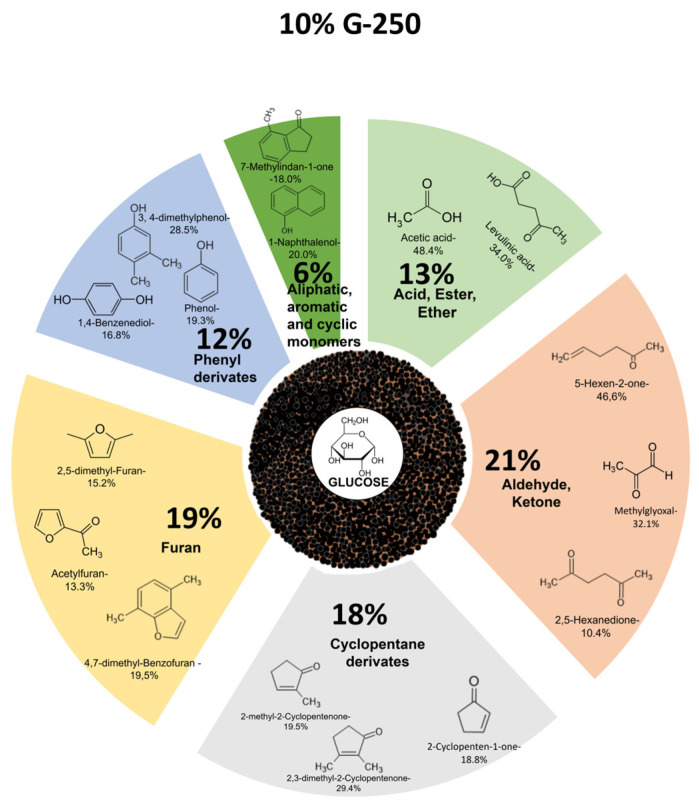

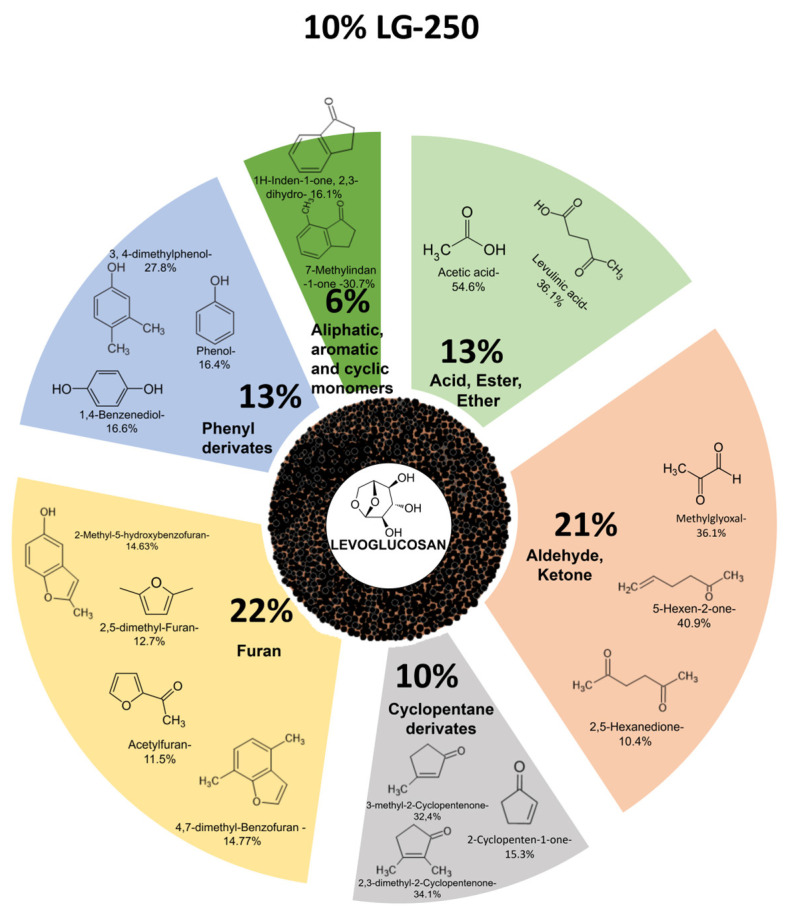
Scheme of the composition of organic matter in biocoal hydrochar (unidentified compounds 10% G and 15% G).

Figure 11 shows the main differences in the structure of glucose- and levoglucosan-based hydrochars. The only difference is the reduction in the amount of cyclopentanes and furans in the levoglucosan-based hydrochars. However, these changes may have an impact on the formation of thermally carbonized hydrochars.

The higher content of cyclopentane derivatives and the lower formation of furan and phenyl compounds from glucose-derived hydrochar can be attributed to differences in hydrothermal reaction pathways. Glucose undergoes fragmentation, isomerization, and recombination reactions leading to the formation of alicyclic structures, which, upon pyrolysis, produce cyclopentane derivatives. In contrast, levoglucosan preferentially undergoes dehydration and aromatization pathways, resulting in hydrochar enriched in furanic and aromatic structures that yield higher amounts of furan and phenyl derivatives during Py-GC/MS analysis.

## 3. Materials and Methods

Two precursors were used for hydrothermal synthesis (HTC)–glucose (G) procured from Sigma-Aldrich (≥99.5%), and levoglucosan (LG) obtained at the Latvian State Institute of Wood Chemistry (≥94%). 1, 10 and 20% feedstock solutions were prepared, which were subsequently treated at 200, 250 and 300 °C for 4 h. Which respective sample names are 1% G-200, 1% G-250, 1% G-300, 10% G-200, 10% G-250, 10% G-300, 20% G-200, 20% G-250, 20% G-300 for glucose and 1% LG-200, 1% LG-250, 1% LG-300, 10% LG-200, 10% LG-250, 10% LG-300, 20% LG-200, 20% LG-250, and 20% LG-300 in case of levoglucosan.

Carbon, nitrogen and hydrogen content were evaluated using the Vario Macro CHNSO (Elementar Analysensysteme GmbH, Langenselbold, Germany) device. The oxygen content was calculated from the difference of 100%.

SEM images were taken to evaluate the morphology of the material. The hydrochar samples were coated twice with a thin layer of gold using an Emitech K550X sputter coater (Emitech Ltd., Ashford, UK). The coated samples were then examined with a VEGA TS 5136MM scanning electron microscope (TESCAN, Brno, Czech Republic) operating at 15 kV and a magnification of 7000×.

The total acid content was determined by potentiometric titration (Titralab Radiometer) of the liquid with 0.1 M KOH solution.

UHPLC analysis was carried out on a Waters Acquity H-Class system. The column was a Waters Acquity UPLC BEH C18 column (1.7 μ, 50 × 2.1 mm), and the mobile phase consisted of methanol and water with 0.1% formic acid additive in a gradient. Flow rate was 0.35 mL/min. The column temperature was 30 °C. Injection volume was 2 μL. Samples were filtered through a Kinesis nylon syringe filter (0.22 μm pore size) before analysis.

The concentration of glucose in the LG HTC process waters was determined by iodometric titration as described here [23].

Analytical pyrolysis (Py-GC/MS at 500 °C) was performed using a Frontier Lab Micro Double-shot Pyrolyser Py-2020iD (Frontier Laboratories Ltd., Koriyama, Fukushima, Japan) directly coupled with Shimadzu GC/MS—QP 2010 apparatus (Shimadzu Corporation, Kyoto, Japan) with capillary column RTX-1701 (Restek Corporation, Bellefonte, PA, USA), 60 m × 0.25 mm × 0.25 µm film (injector temperature 543 K, ion source 523 K with EI of 70 eV, MS scan range *m*/*z* 15–350, carrier gas helium at the flow rate of 1 mL min^−1^ and the split ratio 1:30). The mass of a sample for these tests (residual moisture content < 1%) was 1.00–2.00 mg. The furnace heating program was as follows: isothermal 1 min at 60 °C, then 6 °C min^−1^ to 270 °C, and finally 270 °C for 10 min. The mass spectrometer was operated in the electron impact mode using 70 eV electron energy.

## 4. Conclusions

Hydrothermal carbonization of glucose and levoglucosan solutions enables the formation of spherical hydrochars, the size and morphology of which depend not only on the processing conditions but also on the nature of the precursor.

During the hydrothermal carbonization of levoglucosan, the conversion pathways can be divided into two main routes: (i) hydrolysis to glucose, followed by dehydration to 5-hydroxymethylfurfural (5-HMF), and (ii) direct dehydration and isomerization to 5-HMF. As a result, nearly twice as much 5-HMF is formed from levoglucosan compared to glucose under similar conditions.

In both cases, 5-HMF subsequently undergoes polymerization, nucleation, and phase separation, leading to the formation of hydrochar particles. However, because levoglucosan follows different reaction pathways than glucose, the resulting hydrochars exhibit distinct chemical compositions.

Analytical pyrolysis results indicate that hydrochars derived from levoglucosan contain approximately half the amount of cyclopentane-type compounds compared to hydrochar derived from glucose. This difference can be attributed to variations in intermediate products formed during hydrolysis, dehydration, and polymerization reactions in the hydrothermal carbonization process. These differences allow the functionality of hydrochars to be modified for further thermal carbonization and use.

## Data Availability

The data presented in this study are available on request from the corresponding author.

## Supplementary Materials

The following supporting information can be downloaded at: https://www.mdpi.com/article/10.3390/molecules31081363/s1, Table S1. Relative content of compounds in the group of acids and esters in the pyrolysis volatile products of glucose and levoglucosan hydrochars at different HTC temperatures (200, 250, 300 °C) and at different precursor concentrations (1%, 10% and 20%); Table S2. Relative content of compounds in the group of aldehyde and ketone in the pyrolysis volatile products of glucose and levoglucosan hydrochars at different HTC temperatures (200, 250, 300 °C) and at different precursor concentrations (1%, 10% and 20%); Table S3. Relative content of compounds in the group of cyclopentane compounds in the pyrolysis volatile products of glucose and levoglucosan hydrochars at different HTC temperatures (200, 250, 300 °C) and at different precursor concentrations (1%, 10% and 20%); Table S4. Relative content of compounds in the group of furan compounds in the pyrolysis volatile products of glucose and levoglucosan hydrochars at different HTC temperatures (200, 250, 300 °C) and at different precursor concentrations (1%, 10% and 20%); Table S5. Relative content of compounds in the group of Phenyl and benzen in the pyrolysis volatile products of glucose and levoglucosan hydrochars at different HTC temperatures (200, 250, 300 °C) and at different precursor concentrations (1%, 10% and 20%); Table S6. Relative content of compounds in the group of aliphatic, aromatic and cyclic monomers in the pyrolysis volatile products of glucose and levoglucosan hydrochars at different HTC temperatures (200, 250, and 300 °C) and at different precursor concentrations (1%, 10% and 20%).
