# Supplementary material for: Insights into Carbon Sphere Formation from Glucose and Levoglucosan During Hydrothermal Carbonisation
Source: Molecules. 2026 Apr 21;31(8):1363. doi: 10.3390/molecules31081363 (PMC13118356; doi:10.3390/molecules31081363)
Supplement: Supplementary file 1 [file molecules-31-01363-s001.zip › molecules-4254363-supplementary.pdf]

Supplement to “Insights into Carbon Sphere Formation from Glucose and Levoglucosan during Hydrothermal Carbonisation” .

Ance Plavniece*, Galina Dobele, Kristine Meile, Vilhelmine Jurkjane, Aivars Zhurinsh

Latvian State Institute of Wood Chemistry, Dzerbenes Str. 27, LV-1006 Riga, Latvia
* Correspondence: ance.plavniece@kki.lv

Table S1. Relative content of compounds in the group of acids and esters in the pyrolysis volatile products of glucose and levoglucosan hydrochars at different HTC temperatures (200, 250, 300 °C) and at different precursor concentrations (1%, 10% and 20%).

	**1%**			**10%**			**20%**			**1%**			**10%**			**20%**		
	**G-200**	**G-250**	**G-300**	**G-200**	**G-250**	**G-300**	**G-200**	**G-250**	**G-300**	**LG-200**	**LG-250**	**LG-300**	**LG-200**	**LG-250**	**LG-300**	**LG-200**	**LG-250**	**LG-300**
**Acid, Ester**	100 %									100 %								
Acetic acid	73.7	59.9	55.5	63.6	48.4	41.2	24.7	18.5	26.1	75.1	68.0	62.3	54.5	44.9	33.4	43.2	21.4	25.2
Propanoic acid	11.3	13.6	13.8	8.4	10.0	12.8	4.3	6.0	8.5	11.7	12.0	15.2	6.1	8.9	8.4	5.1	4.2	9.8
2-Propenoic acid	1.8	3.8	6.3	5.0	3.3		1.5	1.1	0.8	5.0	4.3	6.8	2.0	3.2	3.2	2.0	1.7	4.0
Butanoic acid	5.7	11.4	13.4	1.8	4.2	14.0	2.0	4.4	10.0	ND	ND	ND	ND	ND	ND	ND	ND	ND
Butanoic acid, 3-methyl-	2.2	3.1	6.6	ND	ND	ND	9.2	14.0	11.5	ND	ND	ND	ND	ND	ND	ND	ND	ND
Hexanoic acid	5.1	8.2	4.4	ND	ND	ND	ND	ND	ND	ND	ND	ND	ND	ND	ND	ND	ND	ND
Levulinic acid	ND	ND	ND	21.2	34.0	32.0	58.2	56.1	43.1	5.1	10.0	9.1	36.1	40.3	51.6	49.7	72.7	57.9
Propanoic acid, 2-oxo-, methyl ester	ND	ND	ND	ND	ND	ND	ND	ND	ND	3.0	5.7	6.6	1.2	2.7	3.4	0.0	0.0	3.0

Table S2. Relative content of compounds in the group of aldehyde and ketone in the pyrolysis volatile products of glucose and levoglucosan hydrochars at different HTC temperatures (200, 250, 300 °C) and at different precursor concentrations (1%, 10% and 20%).

	**1%**			**10%**			**20%**			**1%**			**10%**			**20%**		
	**G-200**	**G-250**	**G-300**	**G-200**	**G-250**	**G-300**	**G-200**	**G-250**	**G-300**	**LG-200**	**LG-250**	**LG-300**	**LG-200**	**LG-250**	**LG-300**	**LG-200**	**LG-250**	**LG-300**
**Aldehyde, Ketone**	**100 %**									**100 %**								
Acetone and Methylglyoxal	39.0	42.9	35.3	38.9	32.1	20.4	33.1	18.8	4.3	40.3	28.3	32.3	35.3	36.0	ND	38.7	30.8	17.5
5-Hexen-2-one	43.0	28.7	37.2	43.3	46.6	44.8	39.7	42.7	19.6	34.8	43.3	37.5	38.8	40.9	53.6	39.2	41.5	42.8
Acetaldehyde, hydroxy-	2.3	2.4	6.4	1.0	1.2	3.8	5.1	4.4	2.3	7.5	5.6	6.4	8.1	3.1	7.0	4.4	3.3	5.5
2-Propanone, 1-hydroxy-	4.4	8.4	7.5	2.3	2.3	7.1	2.4	2.2	2.3	3.8	6.3	11.2	2.4	1.5	5.7	1.8	1.6	5.8
Hept-3-yn-2-one	ND	ND	ND	4.0	3.0	ND	3.8	1.3	0.0	3.4	2.3	0.0	3.5	3.4	ND	4.4	2.8	ND
3-Buten-2-one, 3-methyl-	6.1	8.7	6.4	3.0	3.3	4.0	3.4	5.2	1.9	3.0	4.5	3.7	2.7	3.5	4.1	3.4	4.0	3.7
4-Heptenal	0.4	3.4	5.0	1.0	1.5	2.7	1.0	2.3	1.2	0.9	2.2	3.5	1.3	1.1	3.6	0.6	0.8	3.0
2,5-Hexanedione	4.7	5.3	2.2	6.5	10.0	17.3	11.5	23.2	14.6	6.3	7.5	5.4	8.0	10.4	21.6	7.6	15.3	17.1
4-Pentenal, 2-methylene-	ND	ND	ND	ND	ND	ND	ND	ND	ND	ND	ND	ND	ND	ND	4.3	ND	ND	4.5

Table S3. Relative content of compounds in the group of cyclopentane compounds in the pyrolysis volatile products of glucose and levoglucosan hydrochars at different HTC temperatures (200, 250, 300 °C) and at different precursor concentrations (1%, 10% and 20%).

	**1%**			**10%**			**20%**			**1%**			**10%**			**20%**		
	**G-200**	**G-250**	**G-300**	**G-200**	**G-250**	**G-300**	**G-200**	**G-250**	**G-300**	**LG-200**	**LG-250**	**LG-300**	**LG-200**	**LG-250**	**LG-300**	**LG-200**	**LG-250**	**LG-300**
**Cyclopentane derivates**	**100 %**									**100 %**								
Cyclopentanone	5.6	7.4	9.0	2.4	3.6	6.1	0.9	ND	ND	2.8	7.6	14.5	0.9	6.3	11.7	1.3	3.6	10.1
2-Cyclopenten-1-one	19.2	13.8	23.5	13.4	18.8	20.0	2.6	2.2	2.3	10.8	24.4	43.6	5.3	13.6	44.8	5.5	11.0	21.7
2-Cyclopenten-1-one, 2-methyl-	12.1	8.1	9.0	15.7	19.5	16.1	4.7	8.9	8.8	6.7	20.0	26.2	8.3	9.8	42.2	4.0	1.5	1.8
4-Cyclopentene-1,3-dione	ND	ND	ND	2.6	1.9	0.0	0.6	0.7	0.5	0.0	0.0	0.0	2.0	1.3	3.3	2.2	1.9	1.8
Cyclopentanone, 3-methyl-	2.3	2.9	3.5	5.8	1.8	1.4	1.1	0.3	0.8	5.7	5.5	7.8	3.9	1.8	3.3	2.9	1.8	2.3
2-Cyclopenten-1-one, 3-methyl-	24.6	21.2	26.1	20.7	21.6	25.3	6.8	10.4	16.5	12.8	37.5	50.9	7.9	16.9	72.9	6.5	14.6	46.0
2-Cyclopenten-1-one, 2,3-dimethyl-	24.8	20.1	21.9	36.0	29.4	28.8	12.7	13.7	19.9	17.1	40.4	54.2	15.3	23.9	74.2	12.7	26.5	48.4
2-Cyclopenten-1-one, 3-(1-methylethyl)-	7.8	15.5	5.3	3.4	3.2	2.3	70.6	63.7	51.2	3.8	8.1	9.3	1.3	2.4	14.0	1.6	3.9	10.1
Cyclopentanone, 2-(2-methylpropylidene)-	3.7	11.0	1.7	ND	ND	ND	ND	ND	ND	2.8	7.6	14.5	0.9	6.3	11.7	1.3	3.6	10.1
Cyclopentane, 1-methyl-1-(2-methyl-2-propenyl)-	ND	ND	ND	ND	ND	ND	ND	ND	ND	10.8	24.4	43.6	5.3	13.6	44.8	5.5	11.0	21.7

Table S4. Relative content of compounds in the group of furan compounds in the pyrolysis volatile products of glucose and levoglucosan hydrochars at different HTC temperatures (200, 250, 300 °C) and at different precursor concentrations (1%, 10% and 20%).

	**1%**			**10%**			**20%**			**1%**			**10%**			**20%**		
	**G-200**	**G-250**	**G-300**	**G-200**	**G-250**	**G-300**	**G-200**	**G-250**	**G-300**	**LG-200**	**LG-250**	**LG-300**	**LG-200**	**LG-250**	**LG-300**	**LG-200**	**LG-250**	**LG-300**
**Furan**					**100 %**									**100 %**				
Furan	ND	ND	ND	ND	ND	ND	ND	ND	ND	ND	ND	ND	0.8	ND	9.6	0.7	ND	ND
Furan, 2-methyl-	31.9	17.1	ND	21.3	12.3	ND	18.8	ND	ND	30.6	7.0	4.9	20.1	12.5	2.0	21.6	11.4	1.5
Furan, 2,5-dimethyl-	25.9	26.1	8.0	16.8	15.2	3.2	15.3	4.6	3.4	20.4	17.0	8.2	10.8	12.7	3.2	13.1	11.7	2.9
Furan, 2-(2-propenyl)-	ND	ND	ND	ND	ND	ND	ND	ND	ND	0.0	0.0	0.0	1.8	0.6	0.0	1.4	2.1	0.0
2(3H)-Furanone, 5-methyl-	2.0	5.6	8.7	4.1	5.0	5.1	7.3	8.5	8.8	ND	ND	ND	ND	ND	ND	ND	ND	ND
Furfural	2.9	2.9	2.8	8.3	5.6	7.7	1.1	1.9	5.4	1.9	4.4	12.7	3.8	6.2	3.2	13.5	4.9	13.4
2-Propenal, 3-(2-furanyl)-	ND	ND	ND	ND	ND	ND	ND	ND	ND	0.0	0.0	0.0	1.0	1.6	2.9	5.4	6.9	4.1
2(3H)-Furanone, 5-methyl-	ND	ND	ND	ND	ND	ND	ND	ND	ND	3.4	6.2	0.0	4.1	5.2	3.4	1.4	7.6	22.6
Acetylfuran	6.8	16.4	20.5	7.3	13.3	11.8	14.3	19.5	16.0	9.5	10.9	15.2	6.1	11.5	8.7	8.2	13.1	8.8
Benzofuran	ND	ND	ND	0.1	1.0	4.6	0.4	2.2	3.4	0.4	2.5	4.9	0.1	1.1	2.5	0.1	0.4	2.4
2-Furancarboxaldehyde, 5-methyl-	14.3	4.5	2.8	15.2	1.4	ND	2.1	2.5	2.4	15.5	2.8	ND	14.4	0.7	1.6	14.1	1.0	2.2
2(5H)-Furanone, 5-methyl-	4.9	6.3	2.9	6.1	8.2	7.3	12.2	14.2	14.4	3.6	4.4	ND	5.1	6.7	5.7	6.8	10.8	4.5
2(3H)-Furanone, dihydro-5-methyl-	ND	ND	ND	0.6	3.4	ND	7.0	7.5	ND	ND	ND	ND	0.7	3.1	ND	1.6	9.2	4.1
Benzofuran, 3-methyl-	4.6	7.8	18.4	2.7	8.0	17.6	5.9	12.1	14.9	3.9	11.8	18.4	2.0	6.2	11.1	2.5	5.4	9.9
Benzofuran, 4,7-dimethyl-	2.1	4.1	20.1	5.5	19.5	33.2	10.6	19.7	23.8	3.0	25.8	35.7	3.1	14.8	21.0	2.1	8.9	17.3
2,5-Furandione, dihydro-	ND	ND	ND	ND	ND	ND	ND	ND	ND	ND	ND	ND	1.5	2.4	1.8	1.1	1.4	ND
2-Furancarboxaldehyde, 5-(hydroxymethyl)-	ND	ND	ND	ND	ND	ND	ND	ND	ND	3.0	ND	ND	21.8	ND	ND	4.4	ND	ND
2-Methyl-5-hydroxybenzofuran	4.6	9.3	15.9	3.6	7.1	9.5	4.9	7.3	7.4	4.9	7.3	ND	2.9	14.6	23.4	2.0	5.0	6.1

Table S5. Relative content of compounds in the group of Phenyl and benzen in the pyrolysis volatile products of glucose and levoglucosan hydrochars at different HTC temperatures (200, 250, 300 °C) and at different precursor concentrations (1%, 10% and 20%).

	**1%**			**10%**			**20%**			**1%**			**10%**			**20%**		
	**G-200**	**G-250**	**G-300**	**G-200**	**G-250**	**G-300**	**G-200**	**G-250**	**G-300**	**LG-200**	**LG-250**	**LG-300**	**LG-200**	**LG-250**	**LG-300**	**LG-200**	**LG-250**	**LG-300**
**Phenyl and benzyl derivates**	**100 %**									**100 %**								
Benzene, ethenyl-	3.2	1.9	3.1	3.7	3.4	1.7	4.8	5.6	8.6	ND	ND	ND	5.2	3.6	10.9	8.1	6.0	2.8
Phenol	23.7	21.1	18.0	20.9	19.3	19.9	20.0	22.1	19.3	25.6	19.8	21.8	16.3	16.4	16.5	17.2	19.6	19.4
Phenol, 2-methyl-	8.7	8.8	10.6	7.8	9.8	7.2	9.8	9.3	12.6	12.2	8.7	10.5	6.6	8.8	10.4	8.6	10.8	11.6
Phenol, 3- and 4-methyl-	31.6	29.5	25.2	30.4	28.5	32.0	27.8	27.6	27.7	31.5	28.7	27.8	35.8	27.8	25.3	34.3	27.5	29.0
Phenol, 3,4-dimethyl-	5.5	5.0	5.2	4.4	7.1	8.4	6.3	7.4	7.6	4.8	8.0	6.1	3.5	7.8	6.2	2.0	5.5	9.1
Phenol, 2,5-dimethyl-	3.6	6.0	6.1	2.4	3.3	5.2	3.2	4.3	4.9	2.9	3.1	4.7	2.9	3.9	5.0	2.4	2.9	4.8
Phenol, 4-ethyl-	0.9	4.5	2.2	ND	ND	ND	ND	ND	ND	ND	ND	ND	ND	ND	ND	ND	ND	ND
Phenol, 2,6-dimethyl-	4.3	3.4	3.9	4.9	4.8	5.8	5.8	6.3	6.3	2.7	4.1	5.2	4.2	4.6	4.8	3.8	5.4	6.4
1,4-Benzenediol	8.3	12.6	10.7	19.6	16.8	11.4	17.2	12.0	8.0	14.4	15.2	12.2	19.4	16.6	11.4	17.3	17.1	11.0
Benzenemethanol, 3-hydroxy-	10.2	7.2	15.1	6.0	7.0	8.4	5.1	5.3	5.0	5.7	12.3	11.7	6.1	10.6	9.6	6.4	5.3	5.9

Table S6. Relative content of compounds in the group of Aliphatic, aromatic and cyclic monomers in the pyrolysis volatile products of glucose and levoglucosan hydrochars at different HTC temperatures (200, 250, 300 °C) and at different precursor concentrations (1%, 10% and 20%).

	**1%**			**10%**			**20%**			**1%**			**10%**			**20%**		
	**G-200**	**G-250**	**G-300**	**G-200**	**G-250**	**G-300**	**G-200**	**G-250**	**G-300**	**LG-200**	**LG-250**	**LG-300**	**LG-200**	**LG-250**	**LG-300**	**LG-200**	**LG-250**	**LG-300**
**Aliphatic, aromatic and cyclic monomers**	**100 %**									**100 %**								
Pentane, 2,4-dimethyl-	3.5	3.1	0.8	ND	ND	ND	ND	ND	ND	ND	ND	ND	ND	ND	ND	ND	ND	ND
Cyclopentene, 1-(1-methylethyl)-	ND	ND	ND	ND	ND	ND	ND	ND	7.9	ND	ND	ND	ND	ND	ND	ND	ND	ND
2-Dodecene, (Z)-	ND	ND	ND	ND	ND	ND	ND	ND	ND	ND	ND	ND	ND	ND	ND	ND	ND	ND
Octanoic Acid	7.1	9.5	4.3	ND	ND	ND	ND	ND	ND	ND	ND	ND	ND	ND	ND	ND	ND	ND
Cyclohexane, (1-methylethylidene)-	ND	ND	ND	ND	ND	5.5	4.1	7.0	8.0	ND	ND	ND	ND	ND	ND	ND	ND	ND
Tridecane	2.5	4.5	1.4	ND	ND	ND	ND	ND	ND	ND	ND	ND	ND	ND	ND	ND	ND	ND
Tridecene	6.8	4.7	2.1	ND	ND	ND	ND	ND	ND	ND	ND	ND	ND	ND	ND	ND	ND	ND
Tetradecane	ND	3.3	ND	ND	ND	ND	ND	ND	ND	ND	ND	ND	ND	ND	ND	ND	ND	ND
3-Tetradecene, (Z)-	3.4	3.1	1.3	ND	ND	ND	ND	ND	ND	ND	ND	ND	ND	ND	ND	ND	ND	ND
1H-Inden-1-one, 2,3-dihydro-	11.3	11.6	10.4	17.2	16.2	19.7	19.2	19.7	20.3	29.0	15.6	9.2	16.3	16.1	14.3	0.6	14.3	16.3
7-Methylindan-1-one	17.1	24.3	55.4	32.0	43.2	51.2	34.9	41.7	37.8	34.5	48.2	59.6	20.2	43.8	37.9	0.5	16.6	48.7
2,5-Octanedione	ND	ND	ND	12.6	9.8	3.5	18.8	6.6	ND	ND	ND	ND	13.9	10.0	ND	0.4	21.0	ND
1-Indanone, 5,6-dimethyl-	ND	ND	ND	ND	ND	ND	ND	5.7	6.4	ND	ND	4.6	ND	ND	ND	ND	ND	ND
1-Naphthalenol	26.2	6.6	ND	23.4	20.0	14.1	22.9	19.4	19.8	36.5	15.9	7.4	34.1	8.9	8.0	0.8	9.9	9.8
Octadecanoic acid, methyl ester	6.5	8.8	7.5	14.8	10.7	ND	ND	ND	ND	ND	ND	ND	ND	8.2	10.7	0.6	8.6	8.0
n-Hexadecanoic acid	13.1	14.5	13.7	ND	ND	ND	ND	ND	ND	ND	ND	ND	ND	ND	ND	ND	ND	ND
Pentadecanoic acid	2.5	6.1	3.0	ND	ND	ND	ND	ND	ND	ND	ND	ND	ND	ND	ND	ND	ND	ND
Heptanoic acid, propyl ester	ND	ND	ND	ND	ND	ND	ND	ND	ND	ND	ND	ND	ND	ND	16.9	1.6	16.4	8.3
1H-Inden-1-one, 2,3-dihydro-5,7-dimethyl-	ND	ND	ND	ND	ND	ND	ND	ND	ND	ND	ND	3.3	ND	ND	ND	ND	ND	ND
1(2H)-Naphthalenone, 3,4-dihydro-2-methyl-	ND	ND	ND	ND	ND	ND	ND	ND	ND	ND	20.4	11.0	15.5	13.0	2.5	ND	13.2	ND
4-Undecyne	ND	ND	ND	ND	ND	ND	ND	ND	ND	ND	ND	2.0	ND	ND	4.2	ND	ND	ND
1H-Inden-1-one, 2,3-dihydro-2-methyl-	ND	ND	3.0	ND	ND	5.4	ND	ND	8.9	ND	ND	3.0	ND	ND	5.4	ND	ND	8.9
